# Coupled Electrokinetics-Adsorption Technique for Simultaneous Removal of Heavy Metals and Organics from Saline-Sodic Soil

**DOI:** 10.1155/2013/346910

**Published:** 2013-10-22

**Authors:** Salihu Lukman, Mohammed Hussain Essa, Nuhu Dalhat Mu'azu, Alaadin Bukhari

**Affiliations:** ^1^Department of Civil and Environmental Engineering, King Fahd University of Petroleum and Minerals, P.O. Box 8632, Dhahran 31261, Saudi Arabia; ^2^Environmental Engineering Department, University of Dammam, Dammam 31451, Saudi Arabia

## Abstract

In situ remediation technologies for contaminated soils are faced with significant technical challenges when the contaminated soil has low permeability. Popular traditional technologies are rendered ineffective due to the difficulty encountered in accessing the contaminants as well as when employed in settings where the soil contains mixed contaminants such as petroleum hydrocarbons, heavy metals, and polar organics. In this study, an integrated in situ remediation technique that couples electrokinetics with adsorption, using locally produced granular activated carbon from date palm pits in the treatment zones that are installed directly to bracket the contaminated soils at bench-scale, is investigated. Natural saline-sodic soil, spiked with contaminant mixture (kerosene, phenol, Cr, Cd, Cu, Zn, Pb, and Hg), was used in this study to investigate the efficiency of contaminant removal. For the 21-day period of continuous electrokinetics-adsorption experimental run, efficiency for the removal of Zn, Pb, Cu, Cd, Cr, Hg, phenol, and kerosene was found to reach 26.8, 55.8, 41.0, 34.4, 75.9, 92.49, 100.0, and 49.8%, respectively. The results obtained suggest that integrating adsorption into electrokinetic technology is a promising solution for removal of contaminant mixture from saline-sodic soils.

## 1. Introduction

Rapid proliferating industrialization has been recorded in recent decades. One of the major environmental consequences of these progressive achievements is the improper release of elevated amounts of variety of organic and inorganic pollutants into the environment. These pollutants could enter the environment directly as a result of accidents, spills during transportation, and leakage from waste disposal sites, storage sites, industrial facilities, and so forth, thereby contaminating the environment. Cooccurrence of complex chemical mixtures such as total petroleum hydrocarbons (TPH), phenols, heavy metals (such as Cr, Cd, Cu, Zn, Pb, and Hg), radionuclides, and pesticides at remediation sites pose potential dangers to human health and the environment and further complicate the remediation process. Some of the pollutants encountered in contaminated soils may be treated using processes like biodegradation, vapor extraction, chemical oxidation, thermal desorption, and incineration. Satisfactory results may not be obtained when these treatment processes are applied to low-permeability soils or those sites contaminated by mixed contaminants (inorganic and organic wastes) because of the difficulty in accessing the contaminants to uniformly deliver treatment reagents. Therefore, innovative remediation technologies must be developed to study the in situ removal of contaminant mixture from soil to ensure a sustainable environment. This has given birth to an important area of current research in in situ soil remediation technology [1–4]. In situ treatment technologies for contaminated soils and groundwater have been the subject of a great deal of research in the last three decades owing to their attendant advantages: potential lower cost, less environmental disruption, and reduction in worker exposure to hazardous materials [1].

Saline-sodic soils (usually found in arid and semiarid regions) possess high electrical conductivity (>4 dS/m) which prevents the application of appropriate voltage gradient in an electrokinetic study owing to current limitations. In addition, these soils are associated with high pH > 8.2, dominated by 2 : 1 type clay minerals and exchangeable sodium at high levels greater than 15 [5, 6]. These properties make saline-sodic soils to be high acid buffering, alkaline, and very difficult to remediate if they are contaminated with heavy metals due to precipitation concerns in alkaline environment. These extreme soil characteristics pose great difficulty in having such soils remediated from mixed contaminants using electrokinetic-based technique. Despite these challenges posed by the soil characteristics, there is a need to investigate possible remediation of such soils using the integrated electrokinetics-adsorption technique giving its promise in remediating low permeability soils [7]. The usual voltage gradient of 1 V/cm for bench-scale studies when applied to such soils could lead to high electric current flow. This in turn could lead to excessive soil heating, reduction in the soil moisture content, high energy and process fluid consumption, and in some cases higher percentage removal of contaminants. 

It has been observed that contaminated soils do not contain single contaminants. Several pollutants appear in the soil as mixed components. In reality, soil polluted with organic contaminants often contains other contaminants such as heavy metals. The implication of the presence of the different nature of the two contaminant groups is that there may be synergistic or antagonistic effects on their respective removal using electrokinetic remediation technique [8, 9]. Reddy [10] posited that the presence of mixed contaminants will retard individual contaminant migration and removal. Also, as organic pollutants are removed by electroosmotic flow and heavy metals by electromigration, the solubility as well as hydrophobicity disparities between the organic pollutants and heavy metals indicates the complexity of electrokinetic remediation of soils polluted with mixed contaminants. To date, several studies have been conducted using electrokinetics (EK) for mixed contamination [11–14]. Of the several electrokinetic remediation techniques, Lasagna process has been found to yield the best removal efficiency of organic contaminants from soils [15]. The general concept of the Lasagna process is the transportation of contaminants from contaminated soil section into treatment zones using major electrokinetic transport mechanisms (i.e., electroosmosis or electromigration). Once at the treatment zones, the contaminants may be removed from the pore water by sorption, degradation, or immobilization depending on treatment zone design [1, 16–18]. Detailed studies of all previous works on the Lasagna process which span from bench-scale investigations to full field-scale remediation of contaminated soils have been reported elsewhere [1, 7, 16–23]. Lasagna process usually uses activated carbon as the sorbent material to improve the removal of contaminants from contaminated soil [1, 22, 23]. 

The main aim of this study is to investigate the possible application of the coupled electrokinetics-adsorption innovative technique that combines electrokinetics and adsorption using locally produced granular activated carbon (GAC) from date palm pits for remediation of local saline-sodic soil contaminated with mixture of toxic pollutants comprising of petroleum byproduct (kerosene), organic compound (phenol), and heavy metals (Cr, Cu, Cd, Zn, Pb, and Hg).

## 2. Materials and Methods

### 2.1. Characterization

Clay used in this study is a local Saudi Arabian clay from Al-Hassa oasis. The clay pH, moisture content, soil organic matter (SOM), electrical conductivity, surface area, and elemental analysis using scanning electron microscopy (SEM) and X-ray diffraction (XRD) methods were determined according to the protocol outlined in the American Society of Testing and Materials (ASTM) standards [25] and reported elsewhere [24]. These physicochemical properties of the saline-soil are presented in Table 1. The mineralogical composition obtained from the XRD result shows that the soil consists mainly of montmorillonite, quartz, and calcite minerals. Scanning electron microscopy revealed the dominant elements to be O and Si whose percentage compositions are 53.11% and 18.46%, respectively. Other elements present in lesser quantities are Ca (7.09%), Al (6.76%), Fe (5.20%), Na (2.69%), Cl (2.69%), K (2.29%), and Mg (1.73%). The GAC used in the present study was produced locally from date palm pits as described elsewhere [26, 27]. 

### 2.2. Adsorption Testing

Single and competitive adsorption of five heavy metals (Cr, Cd, Cu, Zn, and Pb) was performed to determine the selectivity sequence and to understand the adsorption behavior of these metals under different pH conditions. This is particularly important to this study, because, soil mineralogy affects heavy metal adsorption behavior and selectivity sequence under different pH conditions. Lukman et al. [24] reported the detailed procedures carried out for the competitive adsorption testing. 

### 2.3. Coupled Electrokinetics-Adsorption Study

A total of three bench-scale experiments were performed to investigate the treatability of the contaminant mixture using the coupled electrokinetics-adsorption technique and to understand the operating peculiarities of the saline-sodic soil. Two of the experiments had GAC chambers bracketing the contaminated soil chamber and were operated at voltage gradients of 0.6 V/cm and 1 V/cm. The third one utilized only electrokinetics at voltage gradient of 0.6 V/cm. 

#### 2.3.1. Reactor Design and Experimental Procedures

**Figure 1:**
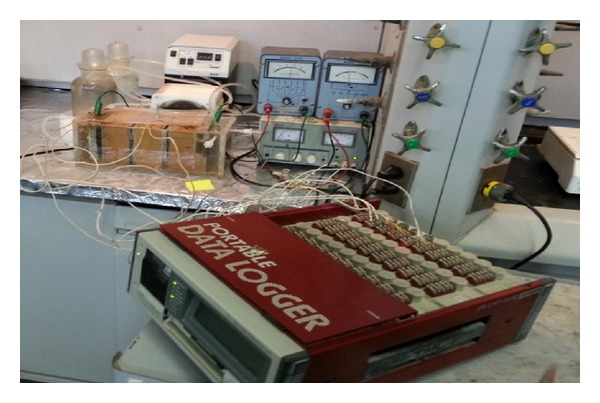
Coupled electrokinetics-adsorption experimental setup.

The Plexiglass reactor total volume was about 2268 cm^3^, made of seven chambers. The overall reactor dimensions are: 24 cm (long) × 10 cm (width) × 12 cm (depth). Approximately 1 kg of local KSA soil was artificially spiked with kerosene, heavy metals (Cu, Cr, Cd, Pb, Zn, and Hg), and phenol at predetermined concentrations. Thorough mixing was done using mechanical mixer (Gilson Company, Inc.) so as to achieve a homogeneous distribution of the contaminants around the soil matrix. The mixed spiked soil was placed in a fume hood for drying over a period of time necessary to evaporate the solvents (hexane and distilled water). Distilled water was added to adjust the final moisture content of the soil to about 33–70%. The initial conditions of the soil pH, moisture content, organic matter, and electrical conductivity were measured as well as the actual initial concentrations of the contaminants. Then, the uniformly mixed contaminated soil was placed into the cell layer by layer. Each layer was compacted with stainless steel spatula, so that the amount of void space was minimized. The Lasagna cell used for the experiments consists of the cell, two graphite electrodes serving as anode and cathode, DC power supply (LG, GP-505), processing fluid reservoirs, heavy duty recirculation pump (BVP Instratec), portable data logger (TDS-303, Tokyo Sokki Kenkyujo Co., ltd) for real-time monitoring of temperature, electric current, and voltage across the system (Figure 1). The two electrode compartments with 240 mL working volume, placed at each end of the cell, were isolated from the soil zone by a porous Perspex plate and filter paper. The conditioning of the electrolyte was controlled by circulation of the anolyte (2 N NaOH) and catholyte (1 N HNO_3_) using pump that is directly attached to the electrode compartments. Two planar-shaped electrodes, 10 cm × 10 cm × 0.5 cm, were used to generate a uniform electric field. Within the described cell, two treatment zones that cut across the cell vertically bracketing the spiked soil compartment were filled with the GAC. The data monitoring system was recording electric current variation, applied voltage, and temperature of the soil compartments on-line following a 30-minute preset time step and automatically stores them for subsequent retrieval using floppy disc which can be read using personal computer for easy data and energy consumption analysis. Treatment without GAC that is using only electrokinetics was conducted so as to ascertain the contribution of the GAC in contaminant removal. The power supply provides a constant DC electric voltage for the electrokinetic test. Every week, fractions of the soil specimens were taken to determine the residual concentrations of the contaminants, soil pH, water content, organic matter, and electrical conductivity. Upon the completion of each test, the electrode assemblies were disconnected, and the soil specimen was extruded from the cell, sectioned into parts, weighed, and preserved in glass vials for organic extraction, heavy metal digestion, and subsequent analyses using the analytical procedures outlined below.

#### 2.3.2. Analytical Procedures for Contaminant Extraction and Analysis

*Heavy Metals.* Extraction of heavy metals from soil samples was performed according to guidelines spelt out in EPA Method 3050B for acid digestion of soils, sediments, and sludges [28] and analyzed using flame atomic absorption spectrometry (AAnalyst 700, PerkinElmer). All soil samples were extracted in duplicates. EPA Method 7000B [29] was employed for heavy metal analysis using flame atomic absorption spectrometry except for mercury which was analyzed using mercury analyzer (Solid Mercury Analyzer SMS 100, Perkin Elmer) according to EPA Method 7473 [30]. 

*Kerosene and Phenol.* A mixture of methylene chloride and hexane (1 : 1) (v/v) was used as the extraction solvent. Soil samples were extracted using pressurized fluid extraction according to EPA Method 3545 procedures [31] using accelerated solvent extractor (ASE 200, Dionex). No separate extraction procedure is required for phenol, because, it will also be extracted along with kerosene from the soil sample. Volume of extract generated was then injected into the GC-MS (Clarus 580, PerkinElmer) equipped with autosampler for analysis. TPH quantification was done by using the total chromatographic area counts using retention time range for the elution of hydrocarbon within the kerosene range C_8_–C_16_. Guidelines spelt out in EPA Method 8270D [32] for the quantification of semivolatile organics by GC-MS were adhered to.

#### 2.3.3. Testing Program

Overall, three experiments were conducted using an initial intended concentration of 100 mg/kg for each contaminant. Three designations used are EK-GAC-1, EK-GAC-2, and EK for coupled EK, adsorption 1 and 2, and EK, respectively. EK-GAC-1 utilized 1 V/cm and served to produce some preliminary information such as the feasibility of attaining reasonable percentage removal and processing fluid conditioning requirements. EK-GAC-2 and EK were then run simultaneously at 0.6 V/cm to ascertain the preference of using the integrated approach over EK alone. 

## 3. Results and Discussion

### 3.1. Clay and GAC Characteristics

From Table 1, the soil pH (9.00) indicates that it contains appreciable soluble salts capable of undergoing alkaline hydrolysis such as sodium carbonate [6]. The hydrolysis of calcite (CaCO_3_) may be limited by its low solubility, thus producing a pH of about 8–8.2 in soils. In addition, Na^+^ ions do not strongly compete with H^+^ ions for exchange sites as does Ca^+^ ions which are strongly and more tightly held on the soil surface. The inability of the displaced Na^+^ ions to inactivate OH^−^ ions results in increased soil pH, which is usually greater than 8.2. Similarly for soil whose pH is greater than 8.2, its exchangeable sodium percentage is greater than 15 [6]. Presence of calcite coupled with alkaline hydrolysis of sodium carbonate gives high electrical conductivity to the soil (8.62 dS/m). The role of soil organic matter (SOM) in heavy metal adsorption is not to be underrated by its low value (3.26%). This is because of the high specific surfaced area and cation exchange capacity possessed by SOM which may reach up to 800–900 m^2^/g and 150–300 cmol/kg, respectively [33]. The physicochemical properties and morphological characteristics of the clay and GAC are detailed elsewhere [24, 26].

### 3.2. Single and Competitive Adsorption of Heavy Metals on Clay

**Figure 2:**
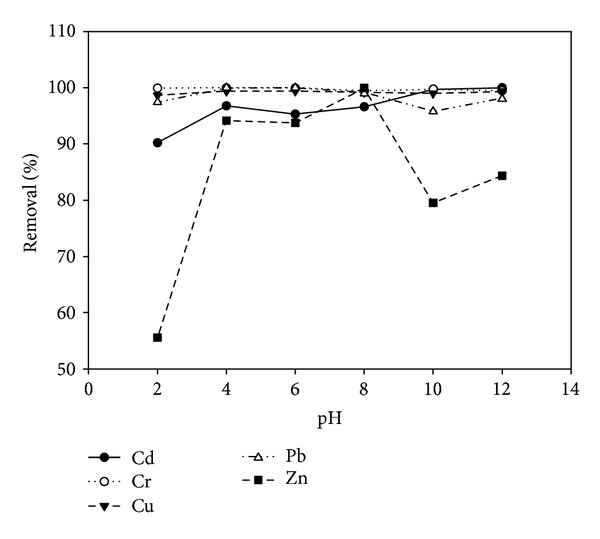
Percentage of heavy metals adsorbed plus any precipitated species at different pH for single component adsorption scenario [24].

**Figure 3:**
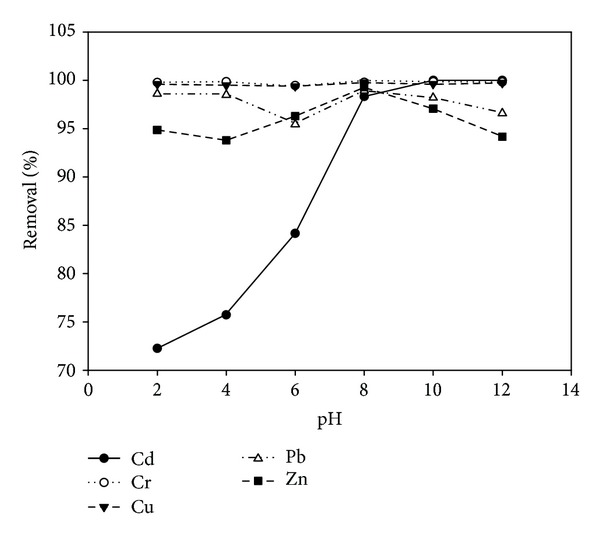
Percentage of heavy metals adsorbed plus any precipitated species at different pH for multicomponent adsorption scenario [24].

**Figure 4:**
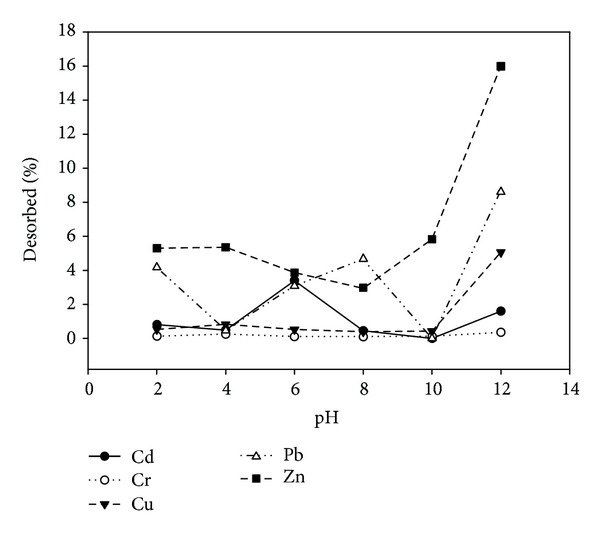
Percentage of heavy metals desorbed plus any precipitated species at different pH for multicomponent desorption scenario [24].

Lukman et al. [24] have found out that the adsorptive capacities of Cu and Zn ions are higher in the multicomponent adsorption scenario than in the single component scenario as presented in Figures 2 and 3. The adsorption selectivity sequences obtained using the coefficient of distribution for the single and multicomponent scenarios are Cr > Pb > Cu > Cd > Zn and Cr > Cu > Pb > Cd > Zn, respectively [24]. Srivastava et al. [34] have reported similar selectivity sequence for the multicomponent scenario. Yong et al. [35] have identified the general factors that influence selectivity sequence to be ionic size or activity, first hydrolysis constant, soil type, and pH of the system. From the multicomponent desorption study presented in Figure 4, it can inferred that trivalent Cr ions were tightly held by the soil surface, thus having the least percentage desorption, followed by Cd and Cu ions. Reddy and his coworkers [36–38] have reported that trivalent Cr ions adsorb highly to soil solids and form cationic species that are insoluble over a wide range of pH. This is in line with the present findings (Figures 3 and 4) which revealed high selectivity for the trivalent Cr during multicomponent adsorption and desorption tests. It may be argued that the solubility of heavy metal ions at alkaline pH is very low due to their precipitation as insoluble hydroxides. However, in this study, the main focus is not on the adsorbed or precipitated species but rather on the mobile or dissolved species that can be removed via electromigration or electroosmotic flow during electrokinetic remediation. Hence, percentage removal (Figures 2 and 3) refers to the amount adsorbed by the soil minerals plus any precipitated metal species. 

### 3.3. Soil pH Distribution during Coupled Electrokinetics-Adsorption Remediation

**Figure 5:**
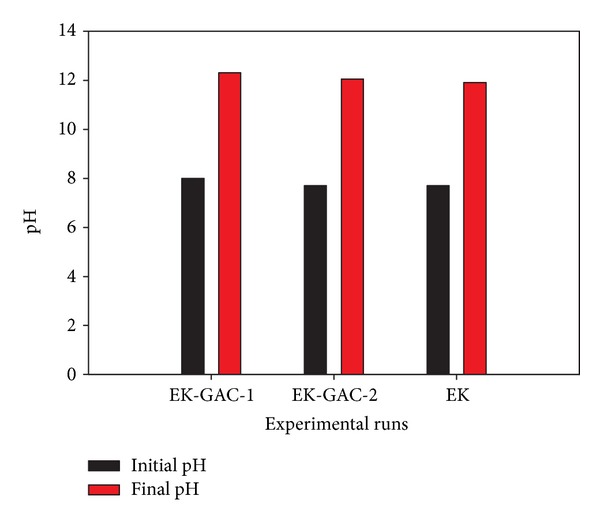
pH variation.

The pH value of the investigated clay is naturally alkaline (pH = 9), which promotes heavy metals precipitation and adsorption onto the clay surface depending on the metal speciation. At the end of the 21-day period, the pH distribution within the soil was found to be approximately 12 (Figure 5) for all the three runs despite dissimilar application of voltage gradients which might be expected to increase the rate of production of H^+^ and OH^−^ radicals and their subsequent migration to the opposite electrodes for higher voltage gradient. This high pH environment might be explained by the presence of calcite in the soil minerals which increases the acid buffering capacity of the soil. It is expected that the carbonates will neutralize the H^+^ ions generated at the anode which suppresses the development and migration of acidic pH front near the anode. Results obtained for electrokinetic remediation of glacial till by Reddy and his coworkers [36] have corroborated this finding. Bipolar effect was also investigated for EK-GAC-2, but pH gradient is not observed; hence, bipolar effect is not present.

### 3.4. Soil Moisture Content, Organic Matter, and Electrical Conductivity

Soil moisture content enhances dissolved contaminant transport by ionic migration and electroosmosis and hence affects removal efficiency. In the present study, the GAC chamber was initially saturated with water, while the spiked soil specimen was kept at an initial moisture content of 33% in each case. This value increased to 52.6, 38.46, and 35.11% for EK-GAC-1, EK-GAC-2, and EK, respectively at the end of the 21-day period. Absence of GAC chambers in the EK run may be responsible for its lowest moisture content at the end of the run. Soil organic matter plays an important role in the adsorption of heavy metal ions even in soils where its value is very low [39]. This is because, SOM possesses very high specific surface area and cation exchange capacity (CEC) which may range between 150 and 300 cmol/kg [33]. The majority of a surface soil's CEC is in fact attributable to its soil organic matter. The initial SOM for the spiked chambers of EK-GAC-1, EK-GAC-2, and EK was 8.22, 6.38, and 6.38%, respectively. At the end of the experiments, these initial values decreased for EK-GAC-1 and EK-GAC-2 and increased slightly for EK. 

**Figure 6:**
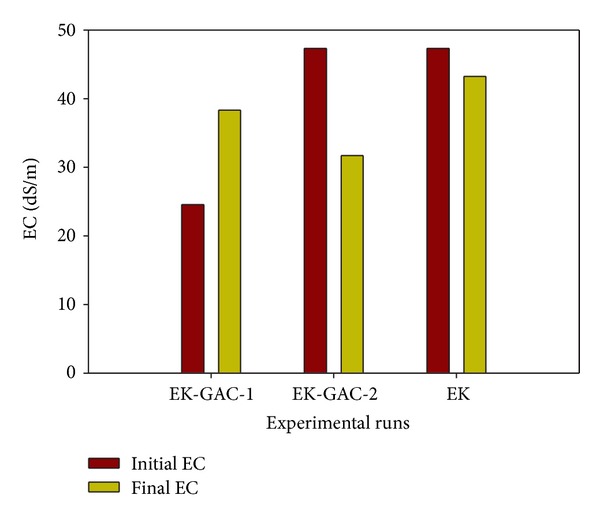
Variation of electrical conductivity.

Soil electrical conductivity (EC) varies with the amount of moisture held by soil particles. Electrical conductivity of clay typically lies between 0.01 and 1 dS/m. Abrol et al. [6] and Sparks [39] have classified soils whose EC and pH are greater than 4 dS/m and 8.2 (at 25°C) to be saline-sodic. The EC of the pristine clay sample is 8.62 dS/m which indicates that it has an excess of dissolved salts which makes it to be classified as saline-sodic soil with exchangeable sodium percentage (ESP) of more than 15. Upon spiking the soil, the EC jumped to 47.3 dS/m due to an increase in the dissolved ions and decreased at the end of the runs to 31.7 and 43.2 dS/m for EK-GAC-2 and EK, respectively (Figure 6). Higher reduction in EC of EK-GAC-2 may be explained by the higher contaminant removal efficiency. In the case of EK-GAC-1, the spiked EC was 24.56 dS/m which increased to 38.3 dS/m at the end of the test. This increase may be attributed to the higher voltage gradient (1 V/cm) used in this test which sped the rate of the electrochemical decomposition of water (at the electrodes) and degradation of the processing fluids.

### 3.5. Variations of Current, Temperature, and Cumulative Electroosmotic Flow

The average electric current recorded for EK-GAC-1, EK-GAC-2, and EK is 0.88, 0.61, and 0.71 A, respectively. Maximum current of 2.8 A was recorded by EK-GAC-1 test and may be attributed to its higher voltage gradient which facilitates faster ionic movement in pore fluid. Dynamic changes in the solution chemistry may be responsible for the observed fluctuating current trend observed in all the tests. Maturi and Reddy (2008) [12] observed somewhat similar current fluctuation. The current values recorded in the tests are 2-3 orders of magnitude higher than those obtained in similar studies employing the Lasagna process and electrokinetics only. This unique and important observation may be explained by the sodicity of the investigated soil which provides large amount of dissolved ions in the pore fluid for effective current conduction. High current flow through the soil may have significant impact on the soil temperature, electroosmotic flow rate, electrode and processing fluid deterioration, removal efficiency, and energy consumption. The average temperature recorded for EK-GAC-2 and EK is 28.5 and 30°C, respectively. For the same tests, the maximum temperature is 34.6 and 40.5°C, respectively. High temperature will reduce the soil moisture content due to pore fluid evaporation and subsequent reduction in current and electroosmotic flow.

Though thermal effects due to temperature rise have not been reported to be significant in bench-scale studies [16], our finding from this study reveals that increasing the voltage gradient more than 1 V/cm leads to considerable rise in the soil temperature which may not be neglected for practical purposes and modeling studies. As such, the general notion of using 1 V/cm for most bench-scale studies needs to be investigated if the soil possesses some properties that were not studied before. Electroosmotic flow is maintained throughout the duration of the tests, EK-GAC-2 and EK tests maintain an average pore volume of 0.75 and 0.66, respectively. Total electroosmotic volumes are 1388 and 1214 mL which are translated into total pore volumes flushed to be 17.35 and 15.17 for EK-GAC-2 and EK tests, respectively. Expectedly, the maximum temperature and electroosmotic flow recorded coincided with the period in which maximum current was recorded. Electroosmotic flow is not influenced by hydraulic gradient in this study as it occurs even under negative hydraulic gradient. Higher electroosmotic flow is expected to occur in the test with higher voltage gradient. It is obvious that the soil zeta potential is not reversed in this study which could reverse the electroosmotic flow. This is because it remains unidirectional throughout the test period.

### 3.6. Contaminant Removal Efficiency

**Figure 7:**
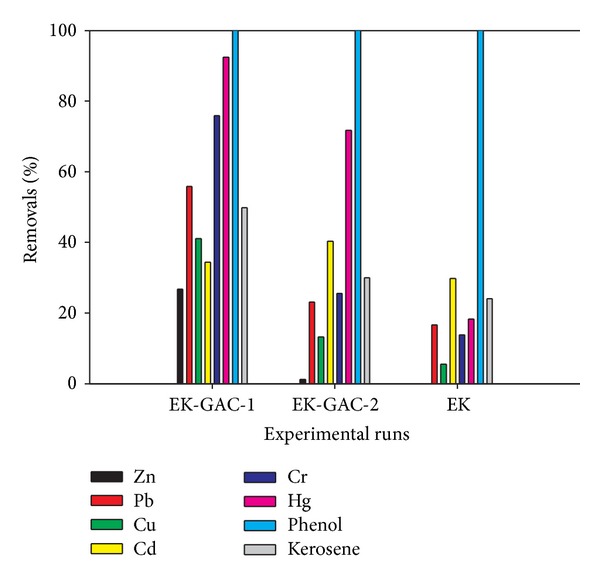
Comparison of the contaminant removal efficiencies for all the tests.

After the operational period of 21 days, significant removal is observed for most of the contaminants in all the tests as presented in Figure 7. Highest and lowest percent removal is observed in phenol and Zn ions, respectively. Only phenol achieved 100% removal possible because it is miscible with water and behaves in the same way as other cationic species [40]. Acar and his coworkers [41] achieved similar percent removal after two pore volumes were flushed. Lukman et al. [24] observed that Zn is the least selective by this soil in competitive aqueous medium, most especially in the alkaline region. From Zn speciation using its Pourbaix diagram [42], it may be said that Zn precipitates as zinc hydroxide at the initial soil pH despite its existence in the form of hydroxocomplexes at pH > 11. Calcareous soils, similar to the one studied here, have been found to perform relatively poor for zinc removal [43]. 

Among the trace elements studied in EK-GAC-1 and EK-GAC-2 tests, Hg removal was highest (92.49%). This may be attributed to the presence of excess Cl^−^ under aerobic conditions and subsequent formation and migration of the mercury complex HgCl_4_^2−^ according to the following reaction [44]:

(1) $$\begin{array}{c} {\text{O}}_{2}+2\text{Hg}+8\text{C}{\text{l}}^{-}+2{\text{H}}_{2}\text{O}\to 2{\text{HgC}{\text{l}}_{4}}^{2-}+4\text{O}{\text{H}}^{-} \end{array}$$

Visual MINTEQ 3.0 was employed to model the Hg speciation before and after the first experimental run (EK-GAC-1) using its dissolved concentration, pH, temperature, and ionic strength. It was assumed that all dissolved Hg species are removed at the end of the experimental run (i.e. 3 weeks).  Table 2 provides the hydroxocomplexes present. The species having the highest initial % of total Hg concentration and removal efficiency is Hg(OH)_2_ (aq) which suggests that precipitated mercury hydroxides can redissolve at high alkaline pH values encountered in this study. The formation of complexes with OH^−^ anions that have increased pore fluid solubility may be attributed to the redissolution of Hg(OH)_2_ (aq) and its subsequent high removal efficiency. Each of the species has been removed by the process, with the dominant removal of Hg(OH)_2_ (aq) corresponding to the actual removal efficiency of Hg during this experimental run (Figure 7).

**Figure 8:**
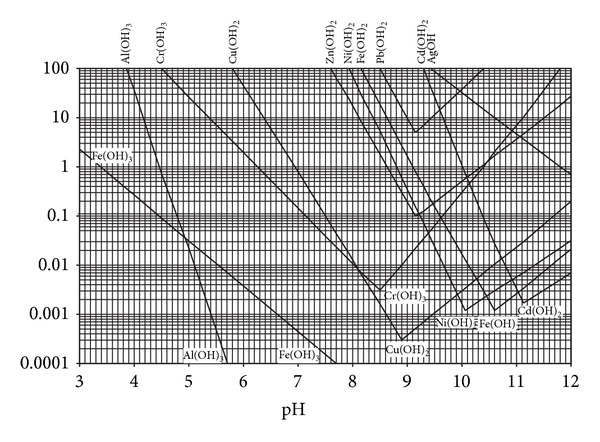
Variation of theoretical solubilities of some heavy metal hydroxides with pH [45].

Figure 8 depicts how precipitated metal hydroxides can redissolve at high pH values. This occurs due to the formation of complexes with OH^−^ anions. These complexes are negatively charged and have increased pore fluid solubility. Consequently, the removal efficiency of these heavy metals is enhanced even under high alkaline condition prevailing in the present study.

Higher electroosmotic flow observed in EK-GAC-1 and EK-GAC-2 tests may be responsible for higher removal of kerosene in these tests than EK test (Figure 7). Generally, introducing GAC chambers in EK-GAC-2 test leads to higher percent removal of all contaminants than the case without the GAC chambers (EK test). In addition, higher voltage gradient produced higher percent removal (EK-GAC-1 and EK-GAC-2 tests).

### 3.7. Conditioning and Energy Consumption

Due to the rapid electrochemical decomposition of water at the electrodes and subsequent generation of H^+^ and OH^−^ ions at the anode and cathode, respectively, there was a need to condition the anode and cathode chambers with H^+^ and OH^−^ neutralizing chemicals. 2 N NaOH and 1 N HNO_3_ were used as the anolyte and catholyte, respectively. Automatic processing fluid recycling was intended, but due to the soil sodicity which led to the passage of high current in the soil, periodic monitoring of the processing fluids pH was necessary. For the EK-GAC-1 test, the catholyte becomes completely basic (pH ≈ 13) after 6 hr, while the anolyte lasted up to 12–18 hr before becoming completely acidic (pH ≈ 0.5). For the EK-GAC-2 and EK tests, the catholyte may last up to 12 hr before it needed replacement. 

Higher current flow in EK test led to higher rate of processing fluids deterioration and higher energy consumption. The total energy consumed per m^3^ of soil treated during the 21- day period is estimated at 8.2, 3.4, and 4 kWhr/m^3^ for EK-GAC-1, EK-GAC-2, and EK tests, respectively.

## 4. Conclusions

The potential of coupling electrokinetics and adsorption using locally produced granular activated carbon from date palm pits for the remediation of natural sodic soil was investigated. The soil was spiked with kerosene, phenol, Cr, Cd, Cu, Zn, Pb, and Hg at given concentrations and three tests (EK-GAC-1, EK-GAC-2, and EK) were run for a period of 21 days. Application of voltage gradient of 1 V/cm to the spiked soil led to high current generation, high electroosmotic flow, high rate of deterioration of processing fluids and anode electrodes, high percent removal, and high energy consumption. Phenol and Zn were found to have the highest and lowest removal efficiency. For the 21 day period of continuous electrokinetics-adsorption experimental run, efficiency for the removal of Zn, Pb, Cu, Cd, Cr, Hg, phenol, and kerosene was found to reach 26.8, 55.8, 41.0, 34.4, 75.9, 92.49, 100.0, and 49.8%, respectively. Despite the high selectivity of trivalent Cr exhibited by the clay soil, the alkaline pH maintained for most of the experimental duration has led to the formation of hydroxocomplexes which were removed due to electromigration. Hence, high percentage removal of the trivalent Cr was recorded. The results obtained suggest that integrating adsorption into electrokinetic technology is a promising solution for removal of contaminant mixture from sodic soils. It is suggested that different types of electrodes should be investigated (for this type of soil) together with the operating parameters (such as polarity reversal rate, pulse and continuous current application) affecting percent removal for simultaneous optimization of the Lasagna process. 

## Figures and Tables

**Table 1:** Physicochemical properties of saline-sodic soil [24].

Property	Value
pH (ASTM D 4972)	9.00
Moisture content, % (ASTM D 2216)	3.91
Soil organic matter, % (ASTM D 2974)	3.26
Hydraulic conductivity, cm/s (ASTM D 5084)	6.91 × 10^−09^
Electrical conductivity, dS/m (ASTM D 1125)	8.62
Average pore width (by BET*), Å	75.64
BET specific surface area, m^2^/g (UOP964)	42.13
Pore volume, cm^3^/g (UOP964)	0.08
Specific gravity (ASTM D 854)	2.77
Liquid limit (ASTM D 4318)	44.71
Plastic limit (ASTM D 4318)	25.95
Plasticity index	18.76
USCS classification (ASTM D 2487)	CL (Clay)

Particle size distribution (ASTM D 422)	
Clay, %	78
Silt, %	6
Very fine sand, %	10
Fine sand, %	5
Medium sand, %	1

*BET: Brunauer-Emmett-Teller.

**Table 2:** Modeling of Hg speciation using Visual MINTEQ 3.0 for EK-GAC-1.

Initial Hg species	Concentration, mol/L	Final Hg species	Concentration, mol/L	% Removed
Hg(OH)_2_ (aq)	4.30*e* − 04	Hg(OH)_2_ (aq)	3.98*e* − 04	92.49
Hg^+2^	9.04*e* − 14	Hg^+2^	1.73*e* − 22	1.91*e* − 07
Hg_2_OH^+3^	4.23*e* − 22	Hg_2_OH^+3^	4.03*e* − 35	9.53*e* − 12
Hg_3_(OH)_3_^+3^	1.79*e* − 22	Hg_3_(OH)_3_^+3^	3.77*e* − 35	2.11*e* − 11
HgOH^+^	2.82*e* − 09	HgOH^+^	1.11*e* − 13	3.93*e* − 03
